# A Bleeding Dieulafoy's Lesion in the Anal Canal

**DOI:** 10.7759/cureus.3853

**Published:** 2019-01-08

**Authors:** Dhineshreddy Gurala, Jobin Philipose, Fady G. Haddad, Deeb Liliane

**Affiliations:** 1 Internal Medicine, Staten Island University Hospital, Northwell Health, Staten Island, USA; 2 Gatsroenterology, Staten Island University Hospital, Northwell Health, Staten Island, USA

**Keywords:** anal, dieulafoy lesion, lower gastrointestinal bleed

## Abstract

Dieulafoy’s lesion (DL) is a dilated aberrant submucosal vessel that erodes through the overlying epithelium in the absence of a primary ulcer. It is a known cause of gastrointestinal (GI) bleeding and commonly located in the lesser curvature of the stomach, but it is rare in the anal canal. We report a unique case of a middle-aged man presenting with lower GI bleeding secondary to an anal DL diagnosed by colonoscopy, managed successfully with endoscopic hemoclips.

## Introduction

Dieulafoy's lesion (DL) represents a tortuous, dilated submucosal artery in the gastrointestinal (GI) tract. It is responsible for 1% to 2% of all cases of acute GI bleeding, though it seems to be under-recognized rather than truly rare [1-3]. The bleeding in this setting can be severe and potentially life-threatening. The stomach is a well-known site for this lesion but it is very uncommon to be found in the anal area [4-5]. We report a case of a middle-aged man with a lower GI bleeding secondary to an anal DL managed with endoscopic hemoclips (ACG abstract: Jobin, Philipose, MD; Fady G. Haddad, MD; Liliane Deeb, MD. Dieulafoy’s Lesion of Ano-Rectum: An Unusual Etiology of Lower Gastrointestinal Bleeding. Program No. P1328. ACG 2018 Annual Scientific Meeting Abstracts).

## Case presentation

A 59-year-old male presented to the emergency room with painless rectal bleeding for five days; his medical history was significant for chronic atrial fibrillation, left atrial thrombus and lower extremity deep vein thrombosis on Warfarin. His family history was unremarkable. He had no previous similar episodes of rectal bleeding, no history of alcohol abuse, smoking or drug abuse, and did not have a fever, abdominal pain or hematemesis.

**Figure 1:**
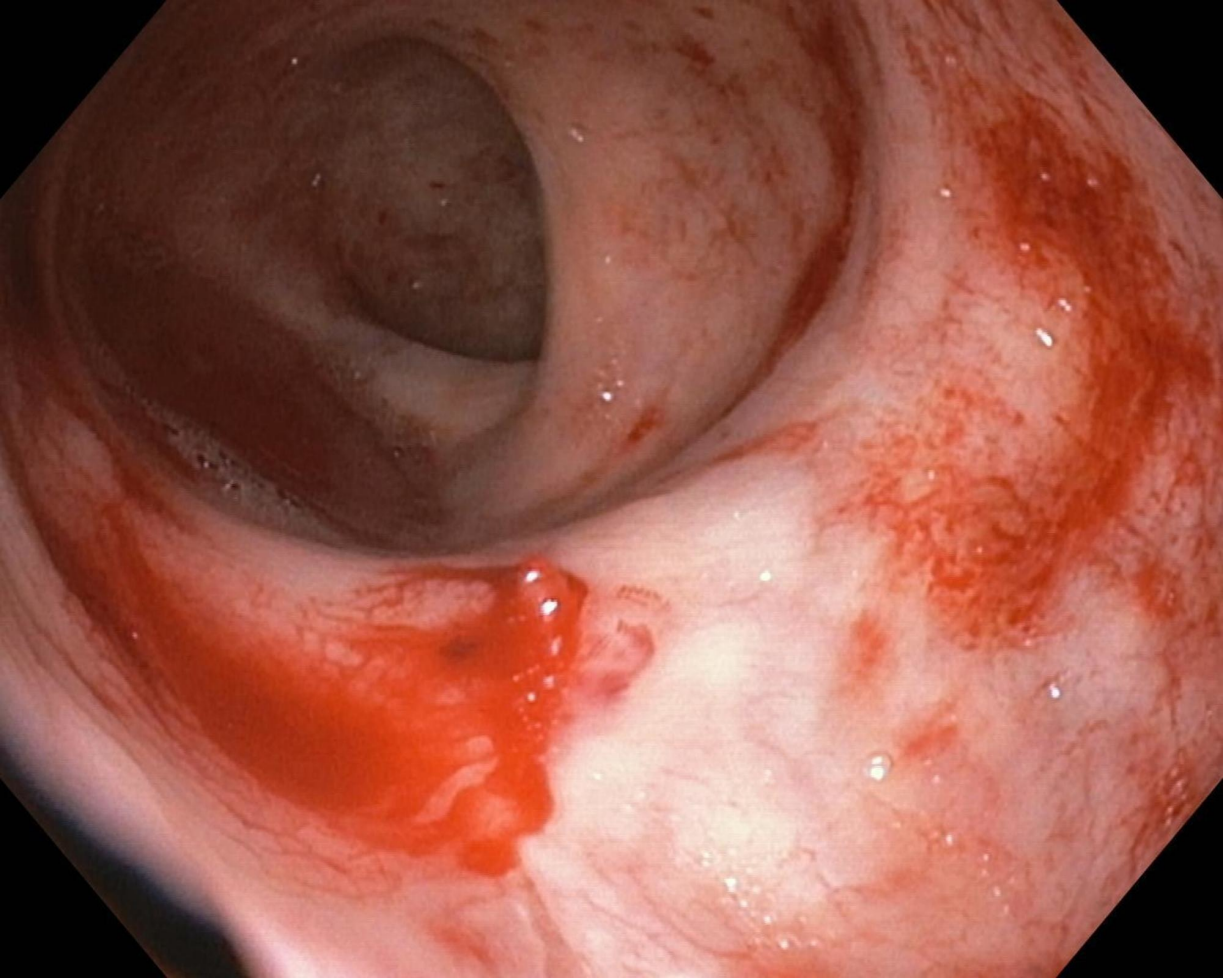
Colonoscopy showing a protruding vessel with active bleeding and normal surrounding mucosa suggestive of Dieulafoy's lesion

**Figure 2:**
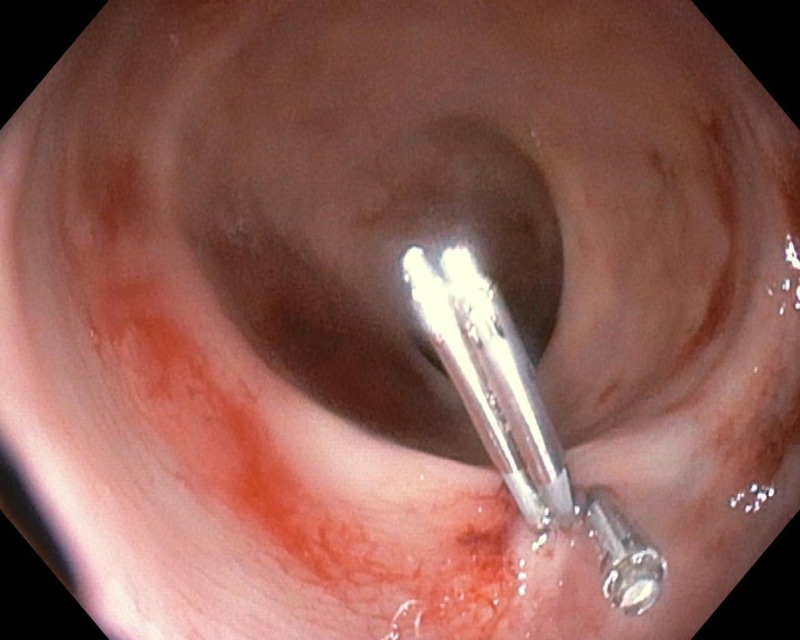
Full control of bleeding after the application of resolution clips in the anal canal

On admission, his blood pressure was 116/80, heart rate of 85 beats per minute, respiratory rate of 18 per minute. On physical exam, he was found to have a small amount of blood in the rectal vault with no external hemorrhoids. Initial laboratory testing revealed white cell count of 7,740 (normal: 4,000-10,000) hemoglobin of 9.8 g/dL (normal: 12-15.5 g/dL), which was lower than high baseline hemoglobin of 13.1 g/dL, platelet count of 132,000 (normal: 130,000-400,000), blood urea nitrogen of 30 mg/dL (normal: 10-20 mg/dL) and creatinine level of 1 mg/dL (normal: 0.7-1.5 mg/dL). Liver enzymes were within normal limits. His international normalized ratio (INR) was 3.9 (normal: 0.65-1.3). The next day, he underwent colonoscopy that demonstrated an actively bleeding protruding vessel in the anal canal just above the dentate line, suggestive of DL (Figure 1; ACG abstract: Jobin, Philipose, MD; Oct 8, 2018). Three endoscopic hemoclips were applied with full control of bleeding (Figure 2; ACG abstract: Jobin, Philipose, MD; Oct 8, 2018). The rest of the rectal mucosa did not show any other bleeding lesion, no inflammation, ulceration, angioectasia or internal hemorrhoids. The patient had no further bleeding afterward. His hemoglobin level remained stable, and he was discharged home three days after presentation.

## Discussion

Gallard first described DL in 1884 as miliary aneurysms of the stomach, more precisely delineated by Sir Paul Georges Dieulafoy who studied three young men who died of gastric hemorrhage in 1898. He termed these lesions as “exulceratio simplex” based on the hypothesis that these were the early stages of peptic ulceration [1,6].

DL is a large penetrating artery that is a histologically normal vessel but does not undergo branching, and hence, the vessel caliber is 1-3 mm, ten times more than that of mucosal capillaries [1-3]. The actual incidence is difficult to determine because of the diagnostic difficulties. The most frequent site is the lesser curvature of the stomach, within 5 cm of esophagogastric junction (70%), although one-third of lesions are extra-gastric including the duodenum (18%), colon (4%), esophagus (8%), small intestine, rectum and rarely anal canal [4-5].

The etiology of these lesions remains unclear. However, they are mainly seen in elderly men with cardiovascular disease, alcohol abuse, diabetes mellitus and kidney disease and those taking non-steroidal anti-inflammatory drugs and warfarin [6-8].

The exact pathophysiology of these lesions is unknown; however, several theories have been proposed. Firstly, pulsations of large vessels disrupt the overlying epithelium that leads to the exposure of vessels to bowel content, resulting in ischemia and rupture. Secondly, gastric wear and tear facilitate thrombosis, leading to subsequent necrosis and hemorrhage. An additional mechanism proposed for colonic DL has been the injury or ulceration of mucosa above the dilated submucosal vessel by solid colon contents, causing its rupture and bleeding. Patients with DL usually present with gastrointestinal bleeding ranging from mild self-limited episodes to massive life-threatening hemorrhage. 

Endoscopy is the primary diagnostic modality, though it can be challenging owing to the small nature of the lesion, concealed in the pool of blood and easily overlooked if it occurs in atypical locations such as in our case. However, initial endoscopy can diagnose DL in 70% [6] of patients but several repeat endoscopies may be required in some patients. Endoscopic criteria for diagnosing DL [2] include: 1) active arterial spurting or micro pulsatile streaming from a mucosal defect <3 mm or through the normal surrounding mucosa or 2) visualization of protruding vessel or 3) appearance of the freshly dense adherent clot with a narrow point of attachment to a minute mucosal defect or a normal-appearing mucosa.

Endoscopic intervention remains the therapeutic modality of choice with the reported success rate exceeding 90%. Recent advances in endoscopic therapy have decreased the mortality from 80% to 8% [9]. Endoscopic treatments used to achieve hemostasis include electrocoagulation, heater probe, laser photocoagulation, sclerotherapy, epinephrine injection, band ligation and through-the-scope and over-the-scope hemoclips. Angiography with gel foam embolization is indicated in patients failing endoscopic therapy, or if the lesion is beyond the reach of the endoscopist. Surgical resection is reserved for 5% of patients who are refractory to endoscopy and angiography [1].

## Conclusions

In conclusion, bleeding DL poses a diagnostic and therapeutic challenge, especially in unusual locations such as an anal canal. Anal DL should be considered as a differential diagnosis of lower gastrointestinal bleed, as we know that traditional diagnoses like hemorrhoids and fissures are almost always favored, leading to potential misdiagnosis with worsened outcomes. 
